# Discerning key parameters influencing high productivity and quality through recognition of patterns in process data

**DOI:** 10.1186/1753-6561-5-S8-P91

**Published:** 2011-11-22

**Authors:** Huong Le, Marlene Castro-Melchor, Christian Hakemeyer, Christine Jung, Berthold Szperalski, George Karypis, Wei-Shou Hu

**Affiliations:** 1 Department of Chemical Engineering and Materials Science, University of Minnesota, Minneapolis, MN 55455, USA; 2 Roche Diagnostics GmbH, 82377 Penzberg, Germany; 3 Department of Computer Science and Engineering, University of Minnesota, Minneapolis, MN 55455, USA

## Background

The adoption of Quality by Design (QbD) approach to biologics manufacturing requires fundamental understanding of complex relationship between the quality of the product, especially critical quality attributes (CQAs), and various parameters of the manufacturing process [1]. This can be approached through multivariate analysis of historical cell culture bioprocess data [2]. In this study, process parameters and raw materials data obtained from 51 runs with final titer varying from 0.8 to 2.0 units and Gal0 glycan ranging from 47.5 to 67.5% was investigated. The aim was to discover prominent patterns which may cause the spread of final process outcome.

## Materials and methods

Offline and online data were processed using linear interpolation and a moving window average method, respectively as described previously [3]. Data from the 1,000 L scale was organized into six cumulative datasets corresponding to days 3, 6, 8, 10, 13, and 15. Euclidean distance for each process parameter between all pairs of runs was calculated and normalized to 0-1. The similarity measure was determined using exponential transformation of the negative of the corresponding distance, and organized into a matrix form. The overall similarity matrix was computed as the weighted combination of all individual similarity matrices. The weight of each reflects how well it correlates to the deviation in final process outcome. A support vector regression (SVR) model was constructed using the overall similarity of all process parameters to predict the final product titer and glycosylation profiles for each cumulative dataset. Prediction accuracy was assessed using the Pearson’s correlation coefficient (r) between the predicted and the actual values.

## Results

Final recombinant antibody concentration (final titer) was predicted with reasonable accuracy using process data at 1,000 L scale. At up to day 3, online and offline data can be indicative of the final titer with an accuracy of r = 46%. Inclusion of data at up to day 6 improved the prediction accuracy markedly to 80%. A modest increase in SVR models predictability was observed when data from day 8 and day 10 was incorporated with r = 83% and 87%, respectively. Online and offline data from days 13 and 15 of the 1,000 L biorectors improved model predictability further to 90%, and 92%, respectively.

Critical process parameters with significant contribution to SVR model predictability were weighted using a non-linear Spearman’s correlation coefficient between its similarity for all pairs of runs and the deviation in their final titer. Parameters with weights (w) greater than 0.2 included stirrer speed, VCD, glucose, LDH, ammonia, and lactate. Each bears significant contribution to prediction of the final titer at different time periods at the 1,000 L scale. Stirrer speed (w = 0.464) appeared to be critical throughout whereas VCD (w = 0.445) and LDH (w = 0.300) became important only after day 3. The contribution of glucose (w = 0.416) and ammonia (w = 0.297) began at day 6, followed by titer values (w = 0.765) at day 8 and lactate (w = 249) at day 10.

Similar results were obtained when Gal0 was used as the objective function for the SVR models in place of the final titer. A marked increase in prediction accuracy was also observed when data from day 6 was included compared to day 3 (from 61% to 85%). After a modest increase to 88% at day 8, almost no further improvement was made using data from later days (10, 13, and 15).

Parameters with high correlation to Gal0 content profile were stirrer speed, VCD, glucose, LDH, ammonia, CO_2_, lactate, temperature, and viability. Among those parameters, six were in common to when final titer was used as the objective function. Three parameters that were critical to prediction of Gal0 but not final titer included CO_2_, temperature, and viability. The time periods in which each parameter had significant correlation to Gal0 content also varies. VCD (w = 0.536), stirrer speed (w = 0.517), and CO_2_ (w = 0.354) were critical from the beginning of the 1,000 L scale. The contribution of LDH (w = 0.417), temperature (w = 0.236), and lactate (w = 0.293) became important at day 3. Day 6 marked the emergence of titer values at previous time points (w = 0.475), glucose (w = 0.424), and ammonia (w = 0.397), followed by viability (w = 0.226) at day 10.

**Figure 1:**
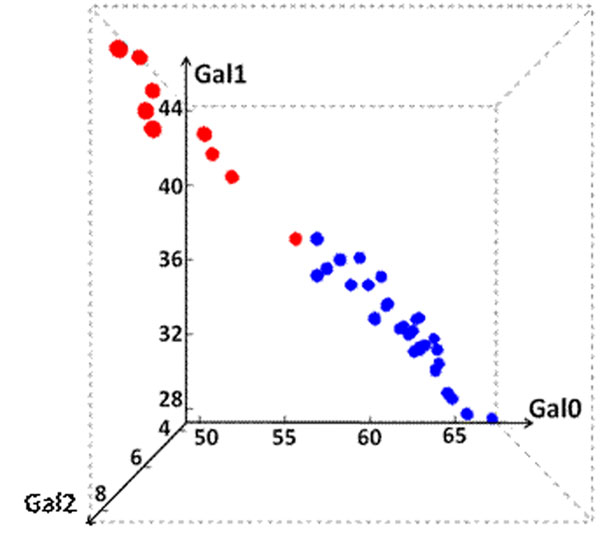
Process runs in three dimensional space of Gal0, Gal1, and Gal2. Low-titer runs are colored in red, and high-titer runs are in blue.

As final titer and Gal0 content were predicted with similar accuracy using similar parameters, a possible relationship between them was explored. Furthermore, other measures of glycosylation profiles such as NG, Gal1, and Gal2 that are possibly relevant to product quality [4] were also included in the analysis. k-means clustering (k = 2) was performed to separate runs into two clusters using each of these measures. Regardless of the measure, the two resulting clusters are reasonably well separated in final titer. Cluster 1 mostly corresponds to high final titer whereas cluster 2 to low final titer. In a three dimensional space of Gal0, Gal1, and Gal2, process runs are also clustered according to their final titer Figure 1). Thus this observation further confirms an intrinsic correlation between product quantity and product quality.

## Conclusions

A strong correlation between productivity (final titer) and product quality (Gal0) was observed. Each was predicted with similarly high accuracy using support vector regression models built upon process data from the 1,000 L bioreactors. Predictability increased significantly when data up to day 6 was analyzed as compared to day 3. Prediction accuracy continued to increase with additional data inclusion but at a slower rate. Several parameters contributed significantly to the deviation of product quantity and quality across runs, including stirrer speed, LDH, lactate, glucose, and VCD. Among those, stirrer speed and VCD appeared to exert the most critical impact on final process outcome from early stages of the 1,000 L scale. This approach represents an important step towards understanding process characteristics for enhanced process robustness, and thus contributes to the advance of bio-manufacturing.
